#  Ultra-processed food consumption and risk of obesity: a prospective cohort study of UK Biobank

**DOI:** 10.1007/s00394-020-02367-1

**Published:** 2020-10-18

**Authors:** Fernanda Rauber, Kiara Chang, Eszter P. Vamos, Maria Laura da Costa Louzada, Carlos Augusto Monteiro, Christopher Millett, Renata Bertazzi Levy

**Affiliations:** 1 Center for Epidemiological Research in Nutrition and Health, University of São Paulo, São Paulo, 01246-904 Brazil; 2 Department of Nutrition, School of Public Health, University of São Paulo, Av. Dr. Arnaldo, 715, São Paulo, 01246-904 Brazil; 3 Public Health Policy Evaluation Unit, School of Public Health, Imperial College London, London, W6 8RP UK; 4 Department of Preventive Medicine, School of Medicine, University of São Paulo, São Paulo, 01246-903 Brazil

**Keywords:** Food processing, Ultra-processed food, Obesity, Cohort study, United kingdom

## Abstract

**Objective:**

The objective of this study was to examine the associations between ultra-processed food consumption and risk of obesity among UK adults.

**Methods:**

Participants aged 40–69 years at recruitment in the UK Biobank (2006–2019) with dietary intakes collected using 24-h recall and repeated measures of adiposity––body mass index (BMI), waist circumference (WC) and percentage of body fat (% BF)––were included (*N* = 22,659; median follow-up: 5 years). Ultra-processed foods were identified using the NOVA classification and their consumption was expressed as a percentage of total energy intake. Multivariable Cox proportional hazards regression models were used to estimate hazard ratios (HR) of several indicators of obesity according to ultra-processed food consumption. Models were adjusted for sociodemographic and lifestyle characteristics.

**Results:**

947 incident cases of overall obesity (BMI ≥ 30 kg/m^2^) and 1900 incident cases of abdominal obesity (men: WC ≥ 102 cm, women: WC ≥ 88 cm) were identified during follow-up. Participants in the highest quartile of ultra-processed food consumption had significantly higher risk of developing overall obesity (HR 1.79; 95% CI 1.06─3.03) and abdominal obesity (HR 1.30; 95% CI 1.14─1.48). They had higher risk of experiencing a ≥ 5% increase in BMI (HR 1.31; 95% CI 1.20─1.43), WC (HR 1.35; 95% CI 1.25─1.45) and %BF (HR 1.14; 95% CI 1.03─1.25), than those in the lowest quartile of consumption.

**Conclusions:**

Our findings provide evidence that higher consumption of ultra-processed food is strongly associated with a higher risk of multiple indicators of obesity in the UK adult population. Policy makers should consider actions that promote consumption of fresh or minimally processed foods and reduce consumption of ultra-processed foods.

**Electronic supplementary material:**

The online version of this article (10.1007/s00394-020-02367-1) contains supplementary material, which is available to authorized users.

## Introduction

Ultra-processed foods, as defined by the NOVA food classification system, are industrial formulations of substances derived from foods, which typically contain cosmetic additives (i.e. flavours and colours) and little, if any, whole foods [1]. Some examples of ultra-processed foods are soft drinks, flavoured dairy drinks, sweet or savoury packaged snacks, confectionery, breakfast cereals, packaged breads and buns, reconstituted meat products and pre-prepared frozen or shelf-stable dishes. These formulations are extremely palatable, convenient, often sold in large portion sizes, and aggressively marketed [2, 3]. The growing production and consumption of ultra-processed products has gradually replaced the traditional food systems and dietary patterns based on minimally processed foods and freshly prepared meals [3, 4]. In a recent global analysis of the trends in sales of ultra-processed foods, the UK has been ranked the third highest consumer of ultra-processed foods (140.7 kg/capita/year) among 80 high- and middle-income countries studied [5].

A growing body of evidence has suggested that the consumption of ultra-processed foods increases the risk of obesity. Analyses of nationally representative dietary surveys conducted in various countries, including the UK, have consistently shown a strong association between consumption of ultra-processed foods and obesogenic dietary nutrient profiles [6–10]. Recent population-based cross-sectional studies have demonstrated a positive association between the consumption of ultra-processed foods and obesity in Brazil [11], the United States [12] and Canada [13]. Furthermore, a two-week, cross-over, randomized controlled trial of 20 weight-stable adults found that higher consumption of ultra-processed foods led to increased energy intake and a substantial gain in body weight and fat mass [14].

There has been limited assessment of the associations between ultra-processed food consumption and obesity using data from prospective cohort studies. A study of 8451 university graduates in Spain found that higher consumption of ultra-processed food was associated with higher risk of developing overweight and obesity within 9 years of follow-up [15]. Another prospective cohort study of 11,827 Brazilian civil servants has found that during an approximately four-year follow-up time, greater ultra-processed food consumption led to larger increases in body mass index (BMI) and waist circumference (WC) [16]. Both studies used food frequency questionnaires to assess dietary intake rather than 24-h recall, that are better at capturing a wider range of foods and beverages consumed, especially those from ultra-processed foods, thus may provide a more precise dietary information. The present study adds to the existing literature by investigating the associations between ultra-processed food consumption and a wider range of obesity indicators—overall and abdominal obesity, changes in BMI, WC and % of body fat—using 24-h dietary recall data as our exposure in a cohort of UK adults.

## Methods

### Data source

The UK Biobank is a large, population-based cohort study [17]. Between 2006 and 2010, 502,536 participants, aged 40–69 years, were recruited and participated in baseline assessments at 22 centres across England, Scotland and Wales. First and second reassessments were carried out between 2012 and 2013, and 2014 and 2019, respectively. During the baseline and each follow-up assessment, participants provided informed consent and completed a self-administered touch-screen questionnaire covering questions on their socio-demographic, lifestyle (e.g. history of smoking and sleep duration) and health-related data. Participants’ physical and anthropometric measurements were collected by trained staffs following standardized procedures. Further details of all measurements can be found in the UK Biobank online protocol (https://www.ukbiobank.ac.uk).

The UK Biobank has received ethical approval from the North West Multi-Centre Research Ethics Committee (16/NW/0274); further details on the scientific rationale, study design and data collection are available elsewhere. Access to the UK Biobank Resource for this research study was granted by the UK Biobank’s Access Sub-Committee under Application Number 29239.

### Dietary assessment

Dietary intakes were collected by a web-based, self-administered questionnaire that aims to record the consumption of over 200 common food and beverage items in the previous 24 h. This 24-h recall was introduced toward the end of the recruitment period (2009–2010). However, all participants with a known email address were invited to complete the questionnaire online at four separate occasions between 2011 and 2012. This web-based questionnaire has been shown to capture similar food and drink items as well as estimated energy and nutrient intakes comparing with an interviewer-administered 24-h recall [18].

The consumption of ultra-processed foods was estimated based on the first 24-h dietary recall of each participant as this best represents their dietary intake at baseline. We classified all food and beverage items according to the NOVA food classification system, which considers the extent and purpose of the food manufacturing process [1]. This classification includes four groups: (1) unprocessed or minimally processed foods, which are natural foods (edible parts of plants or of animals after separation from nature), or natural foods altered by methods, such as freezing, pasteurization, fermentation, grinding and other methods that do not include the addition of substances such as salt, sugar and/or oils or fats (e.g. fresh, dry or frozen fruits or vegetables; grains, flours and pasta; pasteurized or power plain milk, plain yogurt, fresh or frozen meat); (2) processed culinary ingredients, which are substances obtained directly from group 1 foods or from nature via processes that include pressing, refining, grinding, milling and drying, and are consumed in combination with group 1 foods in freshly prepared dishes (e.g. table sugar, oils, butter and salt); (3) processed foods, which are products made by combining group 2 substances (e.g. salt, sugar, oil and fats) with group 1 foods (e.g. vegetables in brine, cheese, simple breads, fruits in syrup and canned fish); and (4) ultra-processed foods—the focus of this study—are food and drink formulations made from salt, sugar, fats and other substances derived from foods but not commonly used as culinary ingredients (such as protein isolates, hydrogenated oils and modified starches) and additives designed to make the final product palatable or more appealing (such as flavours, colours, sweeteners and emulsifiers) (e.g. soft drinks, sweet or savoury packaged snacks, confectionery; packaged breads and buns; reconstituted meat products and pre-prepared frozen or shelf-stable dishes). Detailed description of the NOVA classification can be found elsewhere [1].

For food items with insufficient information for classification purposes, we considered the most frequently consumed alternative (culinary preparation or manufactured product) based on published findings of foods and drinks consumed by the UK population [6]. In case of items for which both options were common, we selected the option with lower level of processing.

The UK Biobank provides the number of portions for each item consumed per day but did not retain the nutritional information (grams and calories) assigned to each food and beverage item. We derived our own estimates by assigning each food and beverage item a typical portion size and an appropriate nutrient profile based on published data for the UK [19, 20]. This allowed us to calculate the nutrients and energy intakes contributed by each of the four NOVA food groups for each individual. We have compared the average total energy intake estimated by us (2067 kcal/day SD 680) with that provided by UK Biobank and found them largely consistent (2138 kcal/day SD 696).

We calculated the relative contribution (as a percentage) to total energy intake for each of the NOVA food groups and subgroups. The dietary contribution of ultra-processed foods is the study exposure and was categorized into quartiles (using sex-specific cut-offs) and a continuous (per 10% increase in consumption) variable.

### Outcomes assessment

Measures of adiposity were BMI, WC and percentage of body fat. Height and weight were measured using a portable stadiometer and weighing scales by trained fieldworkers [21]. BMI was calculated by dividing weight by height in metres squared (kg/m^2^). WC was measured at the midpoint between the iliac crest and the lower rib to the nearest 0.1 cm. Body fat percentage was measured during bioelectrical impedance analysis using a Tanita BC-418 body composition analyser (Tanita Corporation, Arlington Heights, IL, USA) [21]. BMI values of ≥ 30 kg/m^2^ were considered as indicators of overall obesity (referred as obesity hereafter) [22] while WC values of ≥ 102 cm in men and ≥ 88 cm in women as indicators abdominal obesity [23]. Three additional outcomes were derived indicating an increase in BMI, WC or percentage of body fat measurements by 5% or more from baseline to follow-up.

### Covariates

Baseline study covariates included age; sex; quintiles of the Index of Multiple Deprivation (IMD); and level of physical activity (low/moderate/high), current smoking status (smoker/non-smoker), sleep duration (≤ 6 h/day, 7–8 h/day, ≥ 9 h/day), and BMI, WC or BF at baseline adjusted when appropriate. Participant’s physical activity was assessed using self-reported questions that were adapted from the validated short form of the International Physical Activity Questionnaire (IPAQ). This recorded the frequency, duration and intensity of walking, moderate and vigorous activity. We used the previously derived physical activity variable supplied by the UK Biobank that categorized each individual’s physical activity into low/moderate/high based on IPAQ’s data processing guidelines [24]. IMD score is a composite measure of overall deprivation for each small area of the UK and was derived separately for England, Scotland and Wales [25]. Since the IMD scores are updated every 2 years, UK Biobank mapped the participants’ postcodes at recruitment to the same/preceding year of IMD scores published for their corresponding country. We therefore derived the country-specific IMD quintile using the scores provided. The IMD was considered the primary socioeconomic exposure because it was the most complete. However, we also ran models to assess individual-level measure of socioeconomic status (household income).

A very low number of participants had data missing for smoking status (0.03%, *n* = 3) and for sleep duration (0.22%, *n* = 51). These participants were included in the unadjusted analyses. On the other hand, 468 (2.1%) and 3064 (13.5%) participants had missing data on IMD and physical activity variables. To avoid massive exclusion of participants with missing values for IMD and physical activity and risk of selection bias, we included a missing class into the models for these variables.

### Statistical analyses

**Fig. 1:**
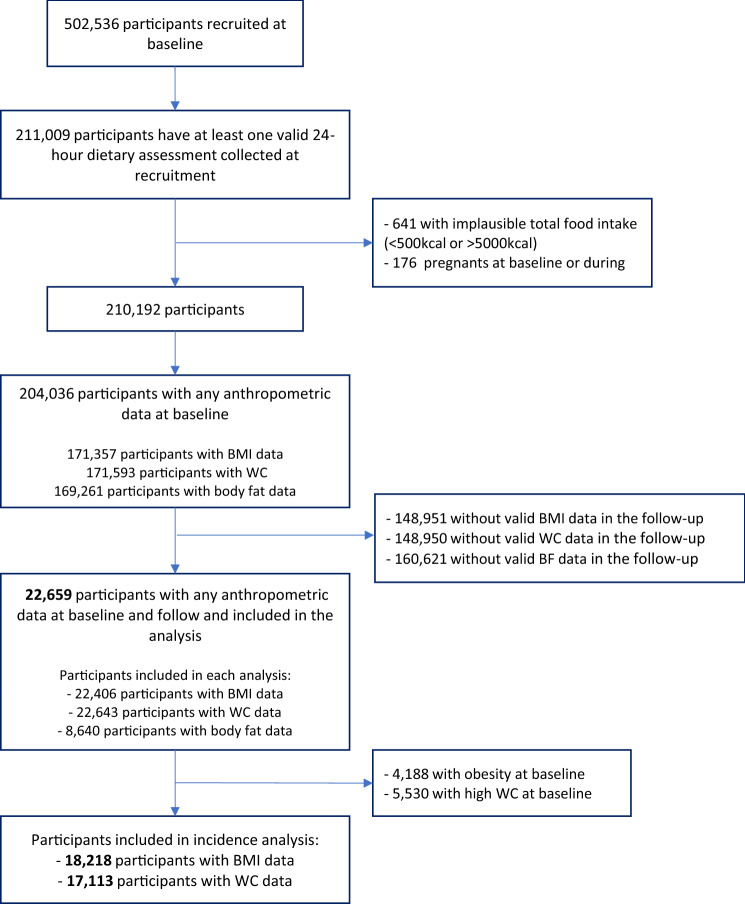
Flowchart for study sample, UK Biobank cohort

For this study, we included participants with a valid 24-h dietary recall collected (*n* = 211,009). We excluded participants with a total energy intake outside of the predefined limits (< 500 kcal and > 5000 kcal) (*n* = 641), women who were pregnant at baseline or became pregnant during the follow-up period (*n* = 176), and participants with missing anthropometric data at baseline or follow-up (*n* = 187,533). Data from 22,659 participants were included for analyses (Fig. 1).

We examined the characteristics of the study population at baseline and by sex-specific quartiles of ultra-processed foods consumption. Group differences by quartiles of ultra-processed foods consumption were assessed using analysis of variance or *χ*^2^ tests where appropriate.

We examined the shape of survival functions between quartiles of ultra-processed food consumption and between subgroups of other covariates using Kaplan–Meier plots, and assessed the equality of survival functions between subgroups using log-rank tests. We used Cox proportional hazards regression models with age as the underlying time metric to estimate the hazard ratios and their corresponding 95% confidence intervals for the incidence of each outcome for every quartile of ultra-processed food consumption considering the lowest quartile as the reference (or as a continuous variable as described above). Participants were followed up until the date when the outcome was identified, or date of their last assessment, whichever occurred first. Our crude model included age (timescale) and quartiles of ultra-processed food consumption. Models were fitted in a stepwise manner: model 1 additionally adjusted for sex and IMD; model 2 additionally adjusted for current smoking status, level of physical activity and sleep duration; and model 3 additionally adjusted for BMI, WC or BF at baseline according to the outcome. The interactions between the exposure variable and some covariates (sex, baseline BMI, baseline WC and baseline BF) were tested by adding a multiplicative term in the Cox regression models. The proportionality assumption of Cox regression model was verified by testing the Schoenfeld residuals against survival time and assessed graphically using plots of − log(− log(survival time)) against log of survival time for each covariate. Linear trend was assessed using the sex-specific quartiles. We verified the assumption of linearity between the consumption of ultra-processed food and risk of obesity indictors using restricted cubic spline functions [26].

We also investigated whether the associations between consumption of ultra-processed foods and risk of obesity differed according to BMI status at baseline (normal weight, overweight and obesity) by stratifying the Cox regression models. To address possible reverse causality bias, we performed a sensitivity analysis, excluding individuals who were on low calorie diet at baseline (3.11%).

All statistical analyses were conducted using Stata version 14.0 and a *p* value of < 0.05 was considered as statistically significant.

## Results

A total of 22,659 participants (52.1% women and 47.9% men) were included in the present study. The analyses of obesity and abdominal obesity incidence were based on 18,218 and 17,113 participants without obesity and abdominal obesity at baseline, respectively. Participants excluded due to missing anthropometric data were similar to those included in this study (Supplementary table S1). The mean baseline age of participants was 55.9 (SD 7.4) years. Table 1 shows the baseline characteristics of participants according to quartiles of the proportion of ultra-processed foods in the diet. Compared with the first quartile (lowest consumption), participants in the highest quartile of ultra-processed foods intake were younger, were more likely to live in the most deprived area and had never smoked, had lower physical activity levels and had higher mean BMI and WC.

**Table 1:** Baseline characteristics of the study population according to sex-specific quartiles of consumption of ultra-processed foods (% of total energy), UK Biobank cohort (n = 22,659)

	All participants	Quartile^a^ of ultra-processed food consumption (% of total energy)				*p* value*
		Means (SD) or *n* (%)				
		1	2	3	4	
Age, years	55.9 (7.4)	56.5 (7.2)	56.4 (7.3)	55.9 (7.4)	55 (7.7)	< *0.001*
Sex, *n* (%)						*1.000*
Female	11,815 (52.1)	2954 (52.1)	2954 (52.1)	2954 (52.1)	2953 (52.1)	
Male	10,844 (47.9)	2711 (47.9)	2711 (47.9)	2711 (47.9)	2711 (47.9)	
Index of multiple deprivation, *n* (%)						< *0.001*
1st quintile (least deprived)	4445 (19.6)	1226 (21.6)	1083 (19.1)	1118 (19.7)	1018 (18.0)	
2nd quintile	4440 (19.6)	1123 (19.8)	1168 (20.6)	1090 (19.2)	1059 (18.7)	
3rd quintile	4448 (19.6)	1137 (20.1)	1120 (19.8)	1087 (19.2)	1104 (19.5)	
4th quintile	4425 (19.5)	1071 (18.9)	1102 (19.5)	1139 (20.1)	1113 (19.7)	
5th quintile (most deprived)	4433 (19.6)	989 (17.5)	1082 (19.1)	1106 (19.5)	1256 (22.2)	
Missing	468 (2.1)	119 (2.1)	110 (1.9)	125 (2.2)	114 (2.0)	
Physical activity, *n* (%)						< *0.001*
Low	3645 (16.1)	865 (15.3)	854 (15.1)	919 (16.2)	1007 (17.8)	
Moderate	8249 (36.4)	2038 (36.0)	2061 (36.4)	2071 (36.6)	2079 (36.7)	
High	7701 (33.9)	2090 (36.9)	1980 (35.0)	1885 (33.3)	1746 (30.8)	
Missing	3064 (13.5)	672 (11.9)	770 (13.6)	790 (14.0)	832 (14.7)	
Smoking status^c^, *n* (%)						< 0.001
Never	13,794 (61.0)	3187 (56.4)	3424 (60.5)	3511 (62.1)	3672 (65.0)	
Previous	7570 (33.5)	2122 (37.6)	1933 (34.2)	1844 (32.6)	1671 (29.6)	
Current	1250 (5.5)	342 (6.1)	300 (5.3)	298 (5.3)	310 (5.5)	
Sleep duration^c^, *n* (%)						*0.493*
≤ 6 h/day	4808 (21.3)	1217 (21.5)	1162 (20.6)	1184 (21.0)	1245 (22.1)	
7–8 h/day	16,367 (72.4)	4085 (72.2)	4139 (73.2)	4111 (72.7)	4032 (71.4)	
≥ 9 h/day	1433 (6.3)	355 (6.3)	351 (6.2)	357 (6.3)	370 (6.6)	
BMI baseline, kg/m^2^	26.7 (4.3)	26.6 (4.2)	26.5 (4.2)	26.6 (4.4)	27 (4.6)	< *0.001*
Obesity at baseline^b^, *n* (%)						< *0.001*
No	18,218 (81.3)	4623 (82.5)	4611 (82.3)	4595 (82.1)	4389 (78.3)	
Yes	4188 (18.7)	979 (17.5)	989 (17.7)	1005 (18.0)	1215 (21.7)	
WC at baseline, cm	88.4 (12.9)	88.3 (12.8)	87.9 (12.7)	88.3 (12.9)	89.2 (13.1)	< *0.001*
Abdominal obesity at baseline^d^, *n* (%)						< *0.001*
No	17,113 (75.6)	4304 (76.0)	4348 (76.8)	4316 (76.2)	4145 (73.2)	
Yes	5530 (24.4)	1357 (24.0)	1311 (23.2)	1346 (23.8)	1516 (26.8)	
Body fat at baseline, %	30.4 (8.4)	30.6 (8.1)	30.1 (8.3)	30.4 (8.6)	30.5 (8.5)	*0.197*

BMI body mass index, *WC*  waist circumference, *BF*  body fat

^a^Sex-specific cut-offs for quarters of ultra-processed food consumption were 24.7%, 41.8%, 54.1% and 71.1% in women and 26.3%, 43.4%, 55.6% and 72.2% in men

^b^Defined as Body Mass Index ≥ 30 kg/m^2^ (World Health Organization, 2003)

^c^A very low proportion of values were missing for smoking status (0.03%, *n* = 3) and for sleep duration (0.22%, *n* = 51)

^d^Defined as waist circumference ≥ 102/88 cm for men and women, respectively (World Health Organization, 2008)

*Analysis of variance or *χ*^2^ test where appropriate

**Fig. 2:**
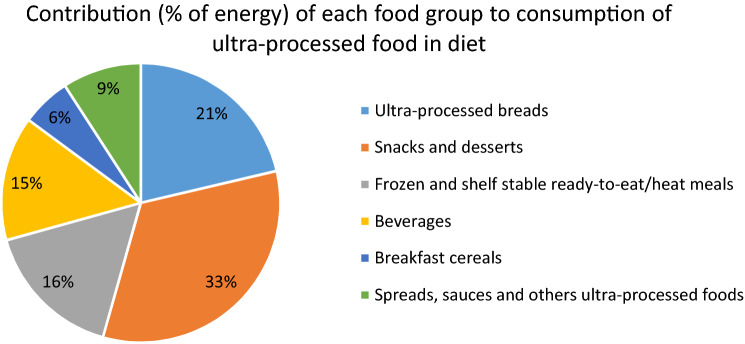
Contribution (% of energy) of each food group to consumption of ultra-processed food in diet. UK Biobank cohort (*n* = 22,659)

The mean contribution of ultra-processed foods to the overall diet (in % of total energy) was 48.6% (SD 18.0) (Supplementary table S2). Briefly, the main food groups contributing to ultra-processed food intake were snacks and desserts (33%, for example, pastries, buns, cakes, biscuits, confectionary, packaged salty snacks and industrial desserts) followed by ultra-processed bread (21%, for example, bagel, burger bun, bread roll and bap), frozen and shelf-stable ready-to-eat/heat meals (16%, for example, industrial chips/French fries, sausage, nuggets, fish fingers and other reconstituted meat products, industrial pizza and packaged pre-prepared meals), beverages (15%, for example, milk-based drinks, soft and fruit drinks, fruit juices, alcoholic drinks and coffee drinks), spreads, sauces and other ultra-processed foods (9%, margarine and other spreads, sauces, dressing and gravies, chocolate/nut spread, spreadable cheese, sweeteners and meat alternative), and breakfast cereals (6%, for example, sweetened cornflake and sweetened oat crunch type cereal) (Fig. 2). Baseline characteristics of the study population included in incidence analysis according to quartiles of consumption of ultra-processed foods are presented in Supplementary table S3. No statistically significant interaction was identified between the dietary contribution of ultra-processed foods and the covariates tested.

The associations between dietary contribution of ultra-processed foods (% of total energy) and indicators of obesity are shown in Table 2. The linearity assumption between intake of ultra-processed food and risks of obesity, abdominal obesity, ≥ 5% increase in BMI, ≥ 5% increase in WC and ≥ 5% increase in BF were assessed using restricted cubic spline (respective *p* values were 0.59, 0.27, 0.03, 0.08 and 0.22) (Supplementary figure S1). No statistically significant violation from the linearity assumption was observed except for the risk of ≥ 5% increase in BMI which showed significance against the null hypothesis (at *α* = 0.05); therefore, we consider the linear relationship as the best approximation for the continuous association between levels of ultra-processed food consumption and risk of each health outcome. A total of 947 cases of obesity and 1900 cases of abdominal obesity were identified during follow-up (97,090 and 91,380 person-years, respectively). After adjustment for potential confounders, participants in the highest quartile of ultra-processed food consumption presented a 79% and 30% relatively higher risk of developing obesity (adjusted HR 1.79; 95% CI 1.06–3.03) and abdominal obesity (adjusted HR 1.30; 95% CI 1.14–1.48), respectively, than those in the lowest quartile of consumption. A 10% increase in the consumption of ultra-processed foods was associated with increased risk of abdominal obesity (adjusted HR 1.06; 95% CI 1.03–1.08), while association for overall obesity was not statistically significant (adjusted HR 1.10; 95% CI 0.99–1.22).

**Table 2:** Association between dietary contribution of ultra-processed food (% of total energy) and indicators of obesity in the UK Biobank cohort

	Ultra-processed food consumption (% of total energy)					
	Sex-specific quarters^a^				*p *for trend^α^	Continuous (10% increase in the consumption)
	HR (95% CI)					
	1	2	3	4		HR (95% CI)
For being obese^b^						
*n* for cases/non-cases	*194/4361*	*231/4324*	*220/4335*	*302/4251*		*947/17,271*
Crude^c^	1	1.21 (1.00–1.46)	1.17 (0.97–1.43)	1.63 (1.36–1.96)	< *0.001*	1.11 (1.07–1.16)
Model 1^c,d^	1	1.19 (0.99–1.45)	1.16 (0.96–1.41)	1.60 (1.33–1.92)	< *0.001*	1.11 (1.07–1.15)
Model 2^c,f^	1	1.21 (1.00–1.47)	1.17 (0.97–1.42)	1.62 (1.35–1.94)	< *0.001*	1.11 (1.07–1.15)
Model 3^c,e^	1	1.50 (0.87–2.58)	1.03 (0.58–1.83)	1.79 (1.06–3.03)	*0.068*	1.10 (0.99–1.22)
For high WC^g^						
*n* for cases/non-cases	*413/3866*	*472/3806*	*483/3795*	*532/3746*		*1900/15,213*
Crude^c^	1	1.16 (1.02–1.33)	1.23 (1.08–1.40)	1.40 (1.23–1.60)	< *0.001*	1.07 (1.04–1.10)
Model 1^c,d^	1	1.16 (1.01–1.32)	1.23 (1.08–1.40)	1.39 (1.22–1.58)	< *0.001*	1.08 (1.05–1.10)
Model 2^c,e^	1	1.16 (1.02–1.33)	1.23 (1.08–1.41)	1.39 (1.22–1.59)	< *0.001*	1.07 (1.04–1.10)
Model 3^c,f^	1	1.17 (1.03–1.34)	1.21 (1.06–1.38)	1.30 (1.14–1.48)	< *0.001*	1.06 (1.03–1.08)
For having a ≥ 5% BMI increase^h^						
*n* for cases/non-cases	*890/4713*	*947/4654*	*917/4684*	*1117/4484*		*3871/18,535*
Crude^c^	1	1.08 (0.98–1.18)	1.06 (0.97–1.17)	1.31 (1.19–1.43)	< *0.001*	-^i^
Model 1^c,d^	1	1.06 (0.97–1.17)	1.06 (0.96–1.16)	1.29 (1.18–1.41)	< *0.001*	-^i^
Model 2^c,e^	1	1.07 (0.98–1.18)	1.07 (0.97–1.17)	1.31 (1.19–1.43)	< *0.001*	-^i^
Model 3^c,f^	1	1.07 (0.98–1.18)	1.07 (0.97–1.17)	1.31 (1.20–1.43)	< *0.001*	-^i^
For having a ≥ 5% WC increase^h^						
*n* for cases/non-cases	*1343/4318*	*1516/4145*	*1510/5151*	*1612/4048*		*5981/16,662*
Crude^c^	1	1.14 (1.06–1.23)	1.18 (1.10–1.27)	1.30 (1.21–1.40)	< *0.001*	1.05 (1.03–1.06)
Model 1^c,d^	1	1.14 (1.06–1.22)	1.18 (1.09–1.27)	1.30 (1.20–1.39)	< *0.001*	1.05 (1.04–1.07)
Model 2^c,e^	1	1.14 (1.06–1.22)	1.18 (1.10–1.27)	1.30 (1.21–1.40)	< *0.001*	1.05 (1.04–1.07)
Model 3^c,f^	1	1.13 (1.05–1.22)	1.18 (1.10–1.27)	1.35 (1.25–1.45)	< *0.001*	1.06 (1.05–1.08)
For having a ≥ 5% BF increase^h^						
*n* for cases/non-cases	*783/1378*	*841/1319*	*836/1324*	*880/1279*		*3340/5300*
Crude^c^	1	1.07 (0.97–1.18)	1.06 (0.96–1.17)	1.13 (1.03–1.24)	*0.023*	1.03 (1.01–1.05)
Model 1^c,d^	1	1.07 (0.97–1.18)	1.06 (0.96–1.17)	1.13 (1.02–1.24)	*0.024*	1.03 (1.01–1.05)
Model 2^c,e^	1	1.08 (0.98–1.19)	1.07 (0.97–1.18)	1.14 (1.04–1.25)	*0.016*	1.03 (1.01–1.05)
Model 3^c,f^	1	1.05 (0.96–1.16)	1.05 (0.95–1.16)	1.14 (1.03–1.25)	*0.014*	1.03 (1.01–1.05)

*BMI* body mass index, *WC*  waist circumference, *BF*  body fat

Mean follow-up times were 5.6 for obesity (97,090 person-years), 5.6 for high waist circumference (91,380 person-years), 5.8 for having a ≥ 5% BMI increase (119,108 person-years), 5.8 for having a ≥ 5% WC increase (121,067 person-years) and 1.8 for having a ≥ 5% body fat increase (17,660 person-years)

^a^Sex-specific cut-offs for quarters of ultra-processed food consumption—ranged from 25.5% of total energy intake (1st quartile) to 71.5% (5th quartile)

^b^Defined as Body Mass Index ≥ 30 kg/m^2^ (World Health Organization, 2003)

^c^Age used as timescale in the Cox models

^d^ Model 1: adjusted for sex and Index of Multiple Deprivation (quintile and missing category)

^e^Model 2: adjusted for Model 1 + physical activity (low, moderate, high and missing category), smoking status (never, previous and current) and sleep duration (≤ 6 h/day, 7–8 h/day, ≥ 9 h/day)

^f^Model 3: adjusted for Model 1 + Model 2 + BMI, WC or BF at baseline (according to the outcome)

^g^Defined as waist circumference ≥ 102/88 cm for men and women, respectively (World Health Organization, 2008)

^h^Participants who had a 5% increase in BMI/WC/body fat from baseline to follow-up

^i^Non-linear association in restricted cubic spline regression

We identified 3871, 5981 and 3340 participants who had a greater than 5% increase in BMI, WC and body fat, respectively (during 119,108, 121,067 and 17,660 person-years of follow-up). After adjustment for potential confounders, participants in the highest quartile of ultra-processed food consumption had significantly higher risk of having a ≥ 5% increase in BMI (adjusted HR 1.31; 95% CI 1.20–1.43), WC (adjusted HR 1.35; 95% CI 1.25–1.45) and body fat (adjusted HR 1.14; 95% CI 1.03–1.25) than those in the lowest quartile of consumption. A significant dose–response relation was observed for all the obesity indicators (*p* for linear trend across quartile < 0.05), except for the incidence of obesity when the model was adjusted for BMI at baseline (*p* for linear trend = 0.068). A 10% increase in the consumption of ultra-processed foods was associated with increased risk of having a ≥ 5% increase in WC (adjusted HR 1.06; 95% CI 1.05–1.08) and body fat (adjusted HR 1.03; 95% CI 1.01–1.05).

The analyses of the association between dietary contribution of ultra-processed food (% of total energy) and indicators of obesity stratified by BMI status at baseline are shown in Table 3. Associations observed among participants who were normal weight or overweight at baseline were similar to those seen for the whole population in the case of a 5% increase in BMI and WC, while no statistically significant association was observed for a 5% increase in the percentage of body fat. For participants who had obesity at baseline, the associations between dietary contribution of ultra-processed food (sex-specific quartile and continuous) and risk of having a ≥ 5% increase in WC and body fat became stronger, while no statistically significant association was observed for a 5% increase in BMI.

**Table 3:** Association between dietary contribution of ultra-processed food (% of total energy) and BMI, waist circumference and body fat according to the BMI status at baseline in the UK Biobank cohort

BMI status at baseline^b^	Ultra-processed food consumption (% of total energy)					
	Sex-specific quarters^a^					Continuous (10% increase in the consumption)
	HR (95% CI)				*p *for trend^α^	
	1	2	3	4		HR (95% CI)
*** ≥ 5% BMI increase ***^***c***^						
Normal weight						
* n *for cases/non-cases	*376/1794*	*372/1792*	*357/1808*	*469/1697*		*1574/7091*
Adjusted model^d^	1	1.02 (0.88 to 1.17)	1.01 (0.87 to 1.17)	1.31 (1.14 to 1.50)	< *0.001*	-^e^
Overweight						
* n *for cases/non-cases	336/2042	*390/1986*	*368/2005*	*443/1925*		*1537/7958*
Adjusted model^d^	1	1.17 (1.01 to 1.35)	1.12 (0.97 to 1.30)	1.39 (1.21 to 1.60)	< *0.001*	-^e^
Obesity						
* n *for cases/non-cases	*182/855*	*176/863*	*169/873*	*216/824*		*743/3415*
Adjusted model^d^	1	0.95 (0.77 to 1.17)	0.92 (0.75 to 1.14)	1.17 (0.96 to 1.43)	*0.145*	-^e^
*** ≥ 5% WC increase***^***c***^						
Normal weight						
* n *for cases/non-cases	*613/1580*	*654/1533*	*647/1540*	*721/1467*		*2635/6120*
Adjusted model^d^	1	1.10 (0.99 to 1.23)	1.14 (1.02 to 1.27)	1.29 (1.15 to 1.43)	< *0.001*	1.05 (1.03 to 1.08)
Overweight						
* n* for cases/non-cases	*538/1860*	*603/1793*	*603/171*	*636/1753*		*2380/7197*
Adjusted model^d^	1	1.10 (0.98 to 1.24)	1.15 (1.02 to 1.29)	1.27 (1.13 to 1.42)	< *0.001*	1.06 (1.03 to 1.08)
Obesity						
* n *for cases/non-cases	*196/851*	*235/815*	*244/807*	*259/791*		*934/3264*
Adjusted model^d^	1	1.21 (1.00 to 1.46)	1.27 (1.05 to 1.54)	1.38 (1.15 to 1.67)	*0.001*	1.07 (1.03 to 1.11)
≥ ***5% BF increase ***^***c***^						
Normal weight						
* n* for cases/non-cases	*361/478*	*405/430*	*383/453*	*414/421*		*1563/1782*
Adjusted model^d^	1	1.15 (0.99 to 1.32)	1.08 (0.93 to 1.24)	1.14 (0.99 to 1.31)	*0.172*	1.02 (0.99 to 1.05)
Overweight						
* n* for cases/non-cases	*326/576*	*330/572*	*329/570*	*346/551*		*1331/2269*
Adjusted model^d^	1	1.01 (0.86 to 1.17)	1.02 (0.87 to 1.19)	1.11 (0.95 to 1.29)	*0.195*	1.03 (0.99 to 1.06)
Obesity						
*n *for cases/non-cases	*89/324*	*96/322*	*125/290*	*125/292*		*435/1228*
Adjusted model^d^	1	1.07 (0.80 to 1.43)	1.37 (1.04 to 1.80)	1.41 (1.07 to 1.86)	*0.004*	1.09 (1.03 to 1.15)

*BMI* body mass index, *WC*  waist circumference, *BF*  body fat

^a^Sex-specific cut-offs for quarters of ultra-processed food consumption

^b^Defined according to World Health Organization cut-offs (WHO, 2003)

^c^Participants who had a 5% increase in BMI/WC/body fat from baseline to follow-up

^d^Adjusted for age (as timescale), sex, Index of Multiple Deprivation (quintile and missing category); physical activity (low, moderate, high, missing category), smoking status (never, previous, and current) and sleep duration (≤ 6 h/day, 7–8 h/day, ≥ 9 h/day)

^e^Non-linear association in restricted cubic spline regression

^α^*p* value for linear trend across quartile of dietary contribution of ultra-processed foods

In the sensitivity analyses, the associations between dietary contribution of ultra-processed food and indicators of obesity were similar after excluding individuals who were on low calorie diet (Supplementary tables S4 and S5). Broadly similar estimates were also found with models using average annual household income instead of IMD score (Supplementary tables S6 and S7).

## Discussion

Findings from this prospective cohort study of British adults show that diets rich in ultra-processed foods were associated with an 79% and 30% significant increase in the risk of obesity and abdominal obesity, respectively. Moreover, higher consumption of ultra-processed foods also increased the risk of a gain in BMI, WC and body fat of 5% or more during the follow-up period (median of 5.6 years). In general, stratification by BMI status at baseline confirmed the findings in the whole population.

Our results corroborate findings from large, population-based cross-sectional studies that identified significant, dose–response associations between higher consumption of ultra-processed foods and obesity [13]. A cohort study of university alumni in Spain confirmed this association by showing a 26% increased risk of developing obesity in the highest quartile of ultra-processed food consumption compared to the lowest quartile [15]. Similarly, a prospective study conducted with Brazilian adults found an approximately 20–30% greater risk of large weight and waist circumference gains in the highest quartile of ultra-processed food consumption compared to the lowest quartile [16].

Several mechanisms may explain the relationship between ultra-processed foods consumption and obesity. Positive associations between ultra-processed foods consumption and the dietary nutrient profiles known to increase the risk of obesity, and other diet-related chronic diseases, are well established in the UK [6, 27] as well as in other middle- and high-income countries [7–10]. A recent 2-week, cross-over, randomized controlled trial of 20 weight-stable healthy adults have shown that, compared to a diet with no ultra-processed foods, consuming a diet high in ultra-processed foods resulted in increases in dietary energy intake as well as substantial body weight and fat gain, even when the ‘ultra-processed diet’ offered to participants was closely matched to the control diet in calories, sugar, fat, sodium, fibre and macronutrients [14]. These findings suggest that characteristics of ultra-processed foods other than their nutrient profile may also be obesogenic. In this same trial, the authors have also found changes in hormone levels, such as appetite-suppressing hormone PYY and hunger hormone ghrelin, and an increase in the eating rate during the ultra-processed diet [14]. In a more recent study based on pooled data from five previously published reports of food energy intake rates, ultra-processed foods had an energy intake rate twice as high as that observed among unprocessed foods (69.4 and 35.5 kcal/min, respectively) [28]. This can be explained by the oro-sensory properties of the ultra-processed foods (e.g. softer food) that make them easier to chew and swallow.

Other potential mechanisms for the link between ultra-processed diets and obesity may be related to the low satiety potential and induction of high glycaemic responses [29] of ultra-processed foods or the little or no presence of intact food matrix in these products which causes changes in the composition and metabolic behaviour of the gut microbiota that promote obesity and other inflammatory diseases [30, 31]. Cosmetic additives, commonly used in the manufacture of ultra-processed foods, could also be part of the mechanisms linking them to obesity. For instance, monosodium glutamate, a flavour enhancer used in several ultra-processed foods, may contribute to obesity by its potential endocrine disrupting effect [32]. Carboxymethylcellulose and polysorbate-80, two emulsifiers that are also commonly present in ultra-processed foods, were shown to induce low-grade inflammation and obesity in mice [33]. In addition, artificial sweetener may contribute to obesity by modulating the gut microbiota [36] and stimulating basal insulin secretion [37]. Finally, ultra-processed foods are typically wrapped in plastic packages, and several plasticizers, such as bisphenol A, have been found associated with obesity [38, 39].

Our study has several strengths. We used data from the UK Biobank, which is a prospective cohort with detailed dietary data collected through a previously validated 24-h recall questionnaire. Of note, the two other cohort studies [15, 16] were based on data collected from food frequency questionnaires, that are less detailed and possibly less accurate for the purpose of categorizing ultra-processed ad non-ultra-processed foods. Another strength is the availability of several measures of adiposity which were objectively measured by trained staff and were not based on self-report. Self-reported data tend to underestimate adiposity, especially among obese women [40]. In addition, we used the NOVA food classification system to classify foods by their level of processing using standardized and objective criteria.

Potential limitations should be considered. First, our study included volunteered subjects who were older and less likely to be obese (18% in our sample vs 29% in British population) than the general population in the UK [41]. They also had higher intakes of unprocessed or minimally processed foods (especially fruit, vegetables and fish) and lower intakes of ultra-processed foods than the British population [6]. Thus, we may have underestimated the association between ultra-processed food consumption and obesity due to a lower contrast between the extreme quartiles of ultra-processed food consumption. Second, a high number of participants were lost to follow-up in this study. Although ultra-processed food consumption was similar between those with and without follow-up data (48.6% and 48.5%, respectively, *p* = 0.260), we cannot preclude the possibility that the relationship between ultra-processed consumption and incidence of overall obesity and abdominal obesity may have differed between groups. Third, a key limitation of the dietary assessment method is underreporting of some foods (particularly unhealthy foods), though 24-h recall questionnaires are recognized to be one of the most comprehensive methods for assessing dietary intake. Previous studies suggest that individuals with obesity may underreport consumption of foods with caloric sweeteners [42], such as desserts and sweet baked goods [43, 44]. This social desirability bias may lead to underestimation of the dietary contribution of ultra-processed foods or dilution of the association between ultra-processed food consumption and adiposity. Nevertheless, accuracy of the dietary intake was improved by online administration of the dietary questionnaire, which is expected to minimize any reporting bias due to social desirability. Moreover, we excluded extreme values of total calorie intake from the analysis. Fourth, the estimation of ultra-processed food consumption based on one 24-h recall as opposed to an averaged intake over multiple days is a limitation. However, due to the inconsistencies in the timing of anthropometric measurements and the administrations of 24-h recalls, we considered the first completed 24-h recall as the best representation of participants’ dietary intake at baseline. Fifth, the Biobank collects limited information indicative of food processing (for example, product brands), which may lead to misclassification of some food items. This bias is more likely to occur in foods, such as pizza, where there is insufficient information available for classification purposes. In such cases, the most frequently consumed alternative (culinary preparation or manufactured product) was chosen. If both options were common, we selected the option with lower level of processing. Potential misclassification would, therefore, lead to an underestimation rather than a spurious exaggeration of ultra-processed food consumption. Sixth, the exact date of the development of the outcomes is not known because the Biobank did not have more frequent measurements. Therefore, we used the date of measurement as an approximation of the date of the event. Finally, due to the observational nature of our study, residual confounding cannot be completely ruled out although we accounted for a wide range of confounders in our statistical models.

Given the endemic presence of ultra-processed foods in the UK (where they contribute to 57% of total calories intake [6]) and globally [5], the analyses presented here suggest that actions to reduce the consumption of ultra-processed foods could produce important public health benefits. Brazil, Uruguay, Ecuador and Peru have already included the avoidance of ultra-processed foods in their food-based dietary guidelines [45–48]. Recently, France has set a goal of 20% reduction in consumption of ultra-processed foods by 2022 [49]. Mexico introduced taxes on common ultra-processed foods, such as sugar-sweetened beverages and high-energy dense snacks, and studies already demonstrated a significant decline in the purchases of those products [50, 51]. In 2016, Chile implemented a law on compulsory warning labels and advertising restrictions for foods/beverages high in at least one critical nutrient, including energy, sugars, saturated fats and sodium, and a recent study has already shown a positive influence of the Chilean law on people’s consumer behaviours [52]. Other ways that governments might intervene to promote freshly prepared meals include subsidies for fresh or minimally processed foods and tax breaks for local food co-operatives and food growers to ensure that healthy foods are affordable and available to all. Ultimately, actions that promote healthy choices of foods and benefits of cooking are the key drivers to improve population health [53].

## Conclusion

This study adds valuable evidence to the literature showing strong associations between ultra-processed food consumption and several measures of adiposity. These findings suggest that policy actions to achieve necessary reductions in ultra-processed food consumption should be considered, such as through adequate food labelling, restrictions on advertising and promotion of these products and fiscal policies that make fresh foods and fresh dishes and meals more affordable than ultra-processed foods. In addition, dietary guidelines for the UK population should be reviewed to take food processing into account.

## Electronic supplementary material

Below is the link to the electronic supplementary material.

Supplementary file1 (DOCX 1955 kb)
