# Supplementary material for: Ultra-processed food consumption and risk of obesity: a prospective cohort study of UK Biobank
Source: Eur J Nutr. 2020 Oct 18;60(4):2169–80. doi: 10.1007/s00394-020-02367-1 (PMC8137628; doi:10.1007/s00394-020-02367-1)
Supplement: Supplementary file 1 — Supplementary file1 (DOCX 1955 kb) [file 394_2020_2367_MOESM1_ESM.docx]

**European Journal of Nutrition**

**Ultra-processed food consumption and risk of obesity: a prospective cohort study of UK Biobank**

**Fernanda Rauber^1,2,3^, Kiara Chang^3^, Eszter P Vamos^3^, Maria Laura da Costa Louzada^1,2^, Carlos Augusto Monteiro^1,2^, Christopher Millett^1,3^, Renata Bertazzi Levy^1,4^**

^1^ Center for Epidemiological Research in Nutrition and Health, University of São Paulo, São Paulo 01246-904, Brazil.

^2^ Department of Nutrition, School of Public Health, University of São Paulo, São Paulo 01246-904, Brazil.

^3^ Public Health Policy Evaluation Unit, School of Public Health, Imperial College London, London W6 8RP, United Kingdom.

^4^ Department of Preventive Medicine, School of Medicine, University of São Paulo, São Paulo 01246-903, Brazil.

**Corresponding author:** Fernanda Rauber, [rauber.fernanda@gmail.com](mailto:rauber.fernanda@gmail.com).

| **Supplementary Table S1. Baseline characteristics of the study population and those individuals excluded due to missing anthropometric data, UK Biobank cohort.** | | | |  |  |
| --- | --- | --- | --- | --- | --- |
|  | **Sample n = 22,659** |  | **Excluded n = 181,377** |  |  |
|  |  |  |  |  |  |
|  | *means (SD) or n (%)* | | |  |  |
| **Age, years** | 55.9 (7.4) |  | 56.1 (8.0) |  |  |
| **Sex, n (%)** |  |  |  |  |  |
| Female | 11,815 (52.1) |  | 100,555 (55.4) |  |  |
| Male | 108,44 (47.9) |  | 80,822 (44.6) |  |  |
| **Index of Multiple Deprivation, n (%)** |  |  |  |  |  |
| 1st quintile (least deprived) | 4445 (19.6) |  | 32,471 (17.9) |  |  |
| 2nd quintile | 4440 (19.6) |  | 32,376 (17.9) |  |  |
| 3rd quintile | 4448 (19.6) |  | 32,455 (17.9) |  |  |
| 4th quintile | 4425 (19.5) |  | 32,365 (17.8) |  |  |
| 5th quintile (most deprived) | 4433 (19.6) |  | 32,412 (17.9) |  |  |
| Missing | 468 (2.1) |  | 19,298 (10.6) |  |  |
| **Physical activity, n (%)** |  |  |  |  |  |
| Low | 3645 (16.1) |  | 28,102 (15.5) |  |  |
| Moderate | 8249 (36.4) |  | 64,909 (35.8) |  |  |
| High | 7701 (33.9) |  | 60,311 (33.3) |  |  |
| Missing | 3064 (13.5) |  | 28,055 (15.5) |  |  |
| **Smoking status, n (%)** |  |  |  |  |  |
| Never | 13,794 (61.0) |  | 101,339 (56.0) |  |  |
| Previous | 7570 (33.5) |  | 64,836 (35.8) |  |  |
| Current | 1250 (5.5) |  | 14,711 (8.1) |  |  |
| **Sleep duration, n (%)** |  |  |  |  |  |
| ≤6h/d | 4808 (21.3) |  | 41,541 (23.0) |  |  |
| 7-8h/d | 16,367 (72.4) |  | 127,449 (70.5) |  |  |
| ≥9h/d | 1433 (6.3) |  | 11,766 (6.5) |  |  |
| **Ultra-processed food (% of total energy)** | 48.61 (0.1) |  | 48.46 (0.0) |  |  |
| **BMI baseline, kg/m^2^** | 26.7 (0.0) |  | 26.9 (0.0) |  |  |
| **WC at baseline, cm** | 88.4 (0.1) |  | 89.0 (0.0) |  |  |
| **Body fat at baseline, %** | 30.2 (0.1) |  | 30.9 (0.0) |  |  |
| BMI = Body mass index; WC = Waist circumference; BF = Body fat | | | |  |  |

| **Supplementary Table S2. Distribution of total energy intake according to NOVA food groups. UK Biobank cohort (n = 22,659)** | | |
| --- | --- | --- |
| **NOVA food groups** | **% of total energy intake** *(mean, SD)* | |
|  | **Total** | |
| **Unprocessed or minimally processed foods** | **35.87** | **16.70** |
| Fruit | 6.85 | 6.04 |
| Cereals | 4.59 | 6.65 |
| Red meat | 4.13 | 6.90 |
| Milk and plain yoghurt | 3.37 | 3.61 |
| Fish and seafood | 2.74 | 6.73 |
| Vegetables | 2.53 | 2.63 |
| Pasta | 2.09 | 5.73 |
| Poultry | 2.35 | 4.79 |
| Fruit juice | 2.17 | 3.28 |
| Roots and tubers | 1.64 | 3.11 |
| Eggs | 1.57 | 3.80 |
| Legumes and nuts | 1.32 | 3.41 |
| Others ^a^ | 0.52 | 1.71 |
| **Processed culinary ingredients** | **2.96** | **4.86** |
| Butter | 2.07 | 4.19 |
| Table sugar | 0.87 | 2.34 |
| Plant oil | 0.02 | 0.37 |
| **Processed foods** | **12.56** | **11.64** |
| Beer and Wine | 6.13 | 8.97 |
| Cheese | 3.00 | 4.50 |
| Processed bread | 1.59 | 5.02 |
| Vegetables and other plant foods preserved in brine | 0.71 | 1.29 |
| Parma ham and other salted, smoked or canned meat or fish | 0.71 | 2.00 |
| Nuts salted | 0.42 | 2.05 |
| **Ultra-processed foods** | **48.61** | **17.98** |
| Ultra-processed breads | 10.36 | 8.17 |
| Pastries, buns, and cakes | 6.89 | 10.18 |
| Milk-based drinks | 4.15 | 8.96 |
| Biscuits | 3.89 | 6.27 |
| Margarine and other spreads | 3.20 | 3.87 |
| Industrial chips (French fries) | 2.80 | 5.83 |
| Sausage and other reconstituted meat products | 2.79 | 6.69 |
| Breakfast cereals | 2.78 | 4.05 |
| Confectionary | 2.28 | 5.05 |
| Soft and fruit drinks and fruit juices | 1.77 | 4.13 |
| Packaged salty snacks | 1.66 | 3.61 |
| Industrial pizza | 1.35 | 7.60 |
| Industrial desserts | 1.32 | 2.82 |
| Packaged pre-prepared meals | 0.99 | 2.48 |
| Sauces, dressing and gravies | 0.86 | 2.33 |
| Alcoholic drink | 0.72 | 2.90 |
| Other ultra-processed foods ^b^ | 0.42 | 1.72 |
| Other beverages and coffee drinks | 0.37 | 1.98 |
| **Total** | **100** |  |
| ^a^ Including coffee + tea + fungi + homemade soup | | |
| ^b^ Including chocolate/nut spread + spreadable cheese + sweeteners + meat alternative | | |

| **Supplementary Table S3. Baseline characteristics of the study population included in incidence analysis according to sex specific quartiles of consumption of ultra-processed foods (% of total energy), UK Biobank cohort.** | | | | | | | | | | | | | |  |  |  |  |  |  |  |  |  |  |  |
| --- | --- | --- | --- | --- | --- | --- | --- | --- | --- | --- | --- | --- | --- | --- | --- | --- | --- | --- | --- | --- | --- | --- | --- | --- |
|  | **Participants included in the BMI incidence analysis** | | | | | |  | **Participants included in the WC incidence analysis** | | | | | | |  |  |  |  |  |  |  |  |  |  |
|  | **All participants** |  | **Quartile^a^ of ultra-processed food consumption (% of total energy)** | | | |  | **All participants (n = 17,113)** |  | **Quartile^a^ of ultra-processed food consumption (% of total energy)** | | | | |  |  |  |  |  |  |  |  |  |  |
|  | **(n = 18,218)** |  | **1** | **2** | **3** | **4** |  |  |  | **1** | **2** | **3** | **4** | |  |  |  |  |  |  |  |  |  |  |
|  | *means (SD) or n (%)* | | | | | |  | *means (SD) or n (%)* | | | | | | |  |  |  |  |  |  |  |  |  |  |
| **Age, years** | 55.9 (7.5) |  | 56.4 (7.2) | 56.3 (7.3) | 55.9 (7.5) | 55 (7.8) |  | 55.7 (7.5) |  | 56.2 (7.3) | 56.1 (7.3) | 55.6 (7.5) | 54.8 (7.8) | |  |  |  |  |  |  |  |  |  |  |
| **Sex, n (%)** |  |  |  |  |  |  |  |  |  |  |  |  |  | |  |  |  |  |  |  |  |  |  |  |
| Female | 9599 (52.7) |  | 2466 (53.3) | 2440 (52.9) | 2419 (52.6) | 2274 (51.8) |  | 8708 (50.9) |  | 2177 (50.9) | 2177 (50.9) | 2177 (50.9) | 2177 (50.9) | |  |  |  |  |  |  |  |  |  |  |
| Male | 8619 (47.3) |  | 2157 (46.7) | 2171 (47.1) | 2176 (47.4) | 2115 (48.2) |  | 8405 (49.1) |  | 2102 (49.1) | 2101 (49.1) | 2101 (49.1) | 2101 (49.1) | |  |  |  |  |  |  |  |  |  |  |
| **Index of Multiple Deprivation, n (%)** |  |  |  |  |  |  |  |  |  |  |  |  |  | |  |  |  |  |  |  |  |  |  |  |
| 1st quintile | 3750 (21.0) |  | 1023 (23.0) | 911 (20.4) | 954 (21.5) | 862 (19.3) |  | 3526 (21.1) |  | 949 (22.7) | 863 (20.6) | 897 (21.5) | 817 (19.5) | |  |  |  |  |  |  |  |  |  |  |
| 2nd quintile | 3633 (20.4) |  | 920 (20.6) | 948 (21.3) | 877 (19.7) | 888 (19.9) |  | 3444 (20.6) |  | 864 (20.6) | 899 (21.5) | 843 (20.2) | 838 (20.0) | |  |  |  |  |  |  |  |  |  |  |
| 3rd quintile | 3592 (20.2) |  | 923 (20.7) | 916 (20.6) | 860 (19.3) | 893 (20.0) |  | 3358 (20.1) |  | 875 (20.9) | 842 (20.1) | 801 (19.2) | 840 (20.0) | |  |  |  |  |  |  |  |  |  |  |
| 4th quintile | 3528 (19.8) |  | 844 (18.9) | 872 (19.6) | 920 (20.7) | 892 (20.0) |  | 3317 (19.8) |  | 791 (18.9) | 819 (19.6) | 864 (20.7) | 843 (20.1) | |  |  |  |  |  |  |  |  |  |  |
| 5th quintile | 3317 (18.6) |  | 747 (16.8) | 810 (18.2) | 837 (18.8) | 923 (20.7) |  | 3098 (18.5) |  | 708 (16.9) | 763 (18.2) | 773 (18.5) | 854 (20.4) | |  |  |  |  |  |  |  |  |  |  |
| **Physical activity, n (%)** | |  |  |  |  |  |  |  |  |  |  |  |  | |  |  |  |  |  |  |  |  |  |  |
| Low | 2612 (14.3) |  | 626 (13.7) | 612 (13.4) | 670 (14.7) | 704 (15.5) |  | 2395 (14.0) |  | 569 (13.3) | 560 (13.1) | 608 (14.2) | 658 (15.4) | |  |  |  |  |  |  |  |  |  |  |
| Moderate | 6667 (36.6) |  | 1636 (35.9) | 1653 (36.3) | 1675 (36.8) | 1703 (37.4) |  | 6188 (36.2) |  | 1534 (35.9) | 1539 (36.0) | 1546 (36.1) | 1569 (36.7) | |  |  |  |  |  |  |  |  |  |  |
| High | 6538 (35.9) |  | 1758 (38.6) | 1676 (36.8) | 1601 (35.2) | 1503 (3.03) |  | 6318 (36.9) |  | 1682 (39.3) | 1612 (37.7) | 1557 (36.4) | 1467 (34.3) | |  |  |  |  |  |  |  |  |  |  |
| Missing | 2401 (13.2) |  | 535 (11.8) | 614 (13.5) | 609 (13.4) | 643 (14.1) |  | 2212 (12.9) |  | 494 (11.5) | 567 (13.3) | 567 (13.3) | 584 (13.7) | |  |  |  |  |  |  |  |  |  |  |
| **Smoking status, n (%)** | | |  |  |  |  |  |  |  |  |  |  |  | |  |  |  |  |  |  |  |  |  |  |
| Never | 11326 (62.3) |  | 2620 (57.6) | 2828 (62.1) | 2863 (63.0) | 3015 (66.4) |  | 10715 (62.7) |  | 2466 (57.8) | 2669 (62.4) | 2734 (64.0) | 2846 (66.7) | |  |  |  |  |  |  |  |  |  |  |
| Previous | 5843 (32.1) |  | 1655 (36.4) | 1478 (32.5) | 1440 (31.7) | 1270 (28.0) |  | 5429 (31.8) |  | 1548 (36.3) | 1380 (32.3) | 1314 (30.8) | 1187 (27.8) | |  |  |  |  |  |  |  |  |  |  |
| Current | 1021 (5.6) |  | 273 (6.0) | 246 (5.4) | 245 (5.4) | 257 (5.7) |  | 942 (5.5) |  | 256 (6.0) | 226 (5.3) | 224 (5.2) | 236 (5.5) | |  |  |  |  |  |  |  |  |  |  |
| **Sleep duration, n (%)** | |  |  |  |  |  |  |  |  |  |  |  |  | |  |  |  |  |  |  |  |  |  |  |
| ≤6h/d | 3672 (20.2) |  | 945 (20.8) | 886 (19.5) | 914 (20.1) | 927 (20.4) |  | 3467 (20.3) |  | 887 (20.7) | 833 (19.5) | 861 (20.2) | 886 (20.8) | |  |  |  |  |  |  |  |  |  |  |
| 7-8h/d | 13411 (73.8) |  | 3331 (73.1) | 3383 (74.5) | 3358 (73.9) | 3339 (73.5) |  | 12626 (73.9) |  | 3140 (73.4) | 3195 (74.9) | 3162 (74.1) | 3129 (73.3) | |  |  |  |  |  |  |  |  |  |  |
| ≥9h/d | 1100 (6.1) |  | 278 (6.1) | 274 (6.0) | 273 (6.0) | 275 (6.1) |  | 989 (5.8) |  | 250 (5.9) | 240 (5.6) | 247 (5.8) | 252 (5.9) | |  |  |  |  |  |  |  |  |  |  |
| **BMI, kg/m^2^** | 25.1 (2.7) |  | 25.1 (2.6) | 25 (2.7) | 25.1 (2.7) | 25.2 (2.7) |  | 25.0 (2.8) |  | 25.0 (2.8) | 24.9 (2.8) | 25.0 (2.8) | 25.2 (2.9) | |  |  |  |  |  |  |  |  |  |  |
| **WC at baseline, cm** | 84.9 (10.4) |  | 85.0 (10.4) | 84.6 (10.5) | 84.8 (10.4) | 85.0 (10.4) |  | 83.7 (9.8) |  | 83.8 (9.7) | 83.5 (9.9) | 83.7 (9.7) | 83.8 (9.7) | |  |  |  |  |  |  |  |  |  |  |
| ^a^ Sex specific cut-off for quarters of ultra-processed food consumption were 24.1%, 41.3%, 53.5% and 70.3% for women and 26.3%, 43.3%, 55.5% and 71.7% for men. | | | | | | | | | | | | | | |  |  |  |  |  |  |  |  |  |  |
| For all covariates except physical activity (13%), a very low proportion of values were missing (≤2%). | | | | | | | | | |  |  |  |  | |  |  |  |  |  |  |  |  |  |  |

**Supplementary Figure S1.** Spline plot for linearity assumption of association between proportion of ultra-processed food in diet and risks of obesity, abdominal obesity, ≥5% increase in BMI, ≥5% increase in WC and ≥5% increase in BF.

| **Supplementary Table S4. Association between dietary contribution of ultra-processed food (% of total energy) and indicators of obesity in the UK Biobank cohort, excluding individuals who were on diet*.** | | | | | | |
| --- | --- | --- | --- | --- | --- | --- |
|  | **Ultra-processed food consumption (% of total energy)** | | | | | |
|  | **Sex-specific quarters ^a^** | | | | | **Continuous (10% increase in the consumption)** |
|  | **1** | **2** | **3** | **4** |  |  |
|  | *HR (95% CI)* | | | | *p for trend^α^* | *HR (95% CI)* |
| **For being obese** ^b^ |  |  |  |  |  |  |
| *n for cases/non-cases* | *188/4244* | *220/4210* | *209/4222* | *284/4146* |  | *901/16,822* |
| Crude ^c^ | 1 | 1.19 (0.98 to 1.44) | 1.15 (0.95 to 1.40) | 1.59 (1.32 to 1.91) | *<0.001* | 1.11 (1.07 to 1.15) |
| Model 1 ^c,d^ | 1 | 1.18 (0.97 to 1.43) | 1.14 (0.93 to 1.38) | 1.55 (1.29 to 1.87) | *<0.001* | 1.10 (1.06 to 1.15) |
| Model 2 ^c,e^ | 1 | 1.20 (0.99 to 1.46) | 1.15 (0.94 to 1.40) | 1.57 (1.30 to 1.89) | *<0.001* | 1.10 (1.06 to 1.15) |
| Model 3 ^c,f^ | 1 | 1.51 (0.85 to 2.69) | 1.00 (0.56 to 1.80) | 1.61 (0.94 to 2.79) | *0.195* | 1.08 (0.97 to 1.20) |
| **For high WC ^g^** |  |  |  |  |  |  |
| *n for cases/non-cases* | *390/3773* | *453/3710* | *456/3707* | *509/3654* |  | *1808/14,844* |
| Crude ^c^ | 1 | 1.19 (1.03 to 1.36) | 1.23 (1.08 to 1.41) | 1.42 (1.25 to 1.62) | *<0.001* | 1.07 (1.04 to 1.10) |
| Model 1 ^c,d^ | 1 | 1.18 (1.03 to 1.35) | 1.23 (1.08 to 1.41) | 1.41 (1.24 to 1.61) | *<0.001* | 1.08 (1.05 to 1.10) |
| Model 2 ^c,e^ | 1 | 1.19 (1.04 to 1.37) | 1.24 (1.08 to 1.42) | 1.42 (1.24 to 1.62) | *<0.001* | 1.08 (1.05 to 1.10) |
| Model 3 ^c,f^ | 1 | 1.20 (1.05 to 1.37) | 1.22 (1.07 to 1.40) | 1.32 (1.16 to 1.51) | *<0.001* | 1.06 (1.03 to 1.09) |
| **For having a ≥5% BMI increase ^h^** |  |  |  |  |  |  |
| *n for cases/non-cases* | *843/4584* | *894/4533* | *867/4560* | *1064/4362* |  | *3668/18,039* |
| Crude ^c^ | 1 | 1.07 (0.97 to 1.18) | 1.06 (0.96 to 1.16) | 1.31 (1.20 to 1.43) | *<0.001* | - ^i^ |
| Model 1 ^c,d^ | 1 | 1.06 (0.96 to 1.16) | 1.05 (0.95 to 1.15) | 1.29 (1.18 to 1.41) | *<0.001* | - ^i^ |
| Model 2 ^c,e^ | 1 | 1.07 (0.97 to 1.18) | 1.06 (0.96 to 1.17) | 1.31 (1.19 to 1.43) | *<0.001* | - ^i^ |
| Model 3 ^c,f^ | 1 | 1.07 (0.97 to 1.18) | 1.06 (0.96 to 1.16) | 1.31 (1.19 to 1.44) | *<0.001* | - ^i^ |
| **For having a ≥5% WC increase ^h^** |  |  |  |  |  |  |
| *n for cases/non-cases* | *1292/4194* | *1459/4025* | *1453/4031* | *1544/3940* |  | *1544/3940* |
| Crude ^c^ | 1 | 1.14 (1.06 to 1.23) | 1.17 (1.09 to 1.27) | 1.29 (1.20 to 1.39) | *<0.001* | 1.05 (1.03 to 1.06) |
| Model 1 ^c,d^ | 1 | 1.14 (1.05 to 1.22) | 1.17 (1.09 to 1.26) | 1.29 (1.19 to 1.38) | *<0.001* | 1.05 (1.04 to 1.07) |
| Model 2 ^c,e^ | 1 | 1.14 (1.05 to 1.22) | 1.17 (1.09 to 1.27) | 1.29 (1.20 to 1.39) | *<0.001* | 1.05 (1.04 to 1.07) |
| Model 3 ^c,f^ | 1 | 1.14 (1.06 to 1.23) | 1.18 (1.09 to 1.27) | 1.34 (1.24 to 1.45) | *<0.001* | 1.06 (1.05 to 1.08) |
| **For having a ≥5% BF increase ^h^** |  |  |  |  |  |  |
| *n for cases/non-cases* | *759/1342* | *822/1278* | *813/1288* | *860/1240* |  | *3254/5148* |
| Crude ^c^ | 1 | 1.08 (0.98 to 1.19) | 1.06 (0.96 to 1.18) | 1.13 (1.03 to 1.25) | *0.023* | 1.03 (1.01 to 1.05) |
| Model 1 ^c,d^ | 1 | 1.08 (0.98 to 1.19) | 1.06 (0.96 to 1.17) | 1.13 (1.03 to 1.25) | *0.025* | 1.03 (1.01 to 1.05) |
| Model 2 ^c,e^ | 1 | 1.08 (0.98 to 1.19) | 1.07 (0.97 to 1.18) | 1.14 (1.03 to 1.26) | *0.016* | 1.03 (1.01 to 1.05) |
| Model 3 ^c,f^ | 1 | 1.05 (0.95 to 1.16) | 1.05 (0.95 to 1.16) | 1.14 (1.03 to 1.25) | *0.017* | 1.03 (1.01 to 1.05) |
| * 705 (3.11%) participants reported they were currently on low calorie diet. | | | | | | |
| BMI = Body Mass Index; WC = waist circumference; BF = Body fat | | | | | | |
| Mean follow-up times were 5.6 for obesity (94,000 person-years), 5.6 for high waist circumference (88,478 person-years), 5.8 for having a ≥5% BMI increase (114,785 person-years), 5.8 for having a ≥5% WC increase (116,697 person-years), and 1.8 for having a ≥5% body fat increase (16,938 person-years). | | | | | | |
| ^a^ Sex specific cut-offs for quarters of ultra-processed food consumption. | | | | | | |
| ^b^ Defined as Body Mass Index ≥30 kg/m^2^ (World Health Organization, 2003). | | | | | |  |
| ^c^ Age used as timescale in the Cox models. | | | | | |  |
| ^d^ Model 1: adjusted for sex and Index of Multiple Deprivation (quintile and missing category). | | | | | |  |
| ^e^ Model 2: adjusted for Model 1 + physical activity (low, moderate, high, missing category), smoking status (never, previous, current), and sleep duration (≤6h/d, 7-8h/d, ≥9h/d); | | | | | | |
| ^f^ Model 3: adjusted for Model 1 + Model 2 + BMI, WC or BF at baseline (according to the outcome); | | | | | |  |
| ^g^ Defined as waist circumference ≥102/88 cm for men and women, respectively (World Health Organization, 2008). | | | | | |  |
| ^h^ Participants who had a 5% increase in BMI/WC/body fat from baseline to follow-up. | | | | | |  |
| ^e^ Non-linear association in restricted cubic spline regression. | | | | | |  |
| ^α^ p value for linear trend across quartile of dietary contribution of ultra-processed foods. | | | | | |  |

| **Supplementary Table S5. Association between dietary contribution of ultra-processed food (% of total energy) and BMI, waist circumference and body fat according to the BMI status at baseline in the UK Biobank cohort, excluding individuals who were on diet*.** | | | | | | |
| --- | --- | --- | --- | --- | --- | --- |
|  | **Ultra-processed food consumption (% of total energy)** | | | | | |
|  | **Sex-specific quarters ^a^** | | | | | **Continuous (10% increase in the consumption)** |
|  | **1** | **2** | **3** | **4** |  |  |
| **BMI status at baseline ^b^** | *HR (95% CI)* | | | | *p for trend^α^* | *HR (95% CI)* |
| **≥5% BMI increase ^c^** | | | | | | |
| **Normal weight** |  |  |  |  |  |  |
| *n for cases/non-cases* | *360/1762* | *357/1761* | *341/1777* | *453/1665* |  | *1512/6965* |
| Adjusted model ^d^ | 1 | 1.02 (0.88 to 1.18) | 1.00 (0.87 to 1.17) | 1.33 (1.15 to 1.52) | *<0.001* | - ^e^ |
| **Overweight** |  |  |  |  |  |  |
| *n for cases/non-cases* | 317/1989 | *366/1934* | *346/1951* | *417/1875* |  | *1446/7744* |
| Adjusted model ^d^ | 1 | 1.17 (1 to 1.36) | 1.12 (0.96 to 1.31) | 1.39 (1.2 to 1.61) | *<0.001* | - ^e^ |
| **Obesity** |  |  |  |  |  |  |
| *n for cases/non-cases* | *167/820* | *167/823* | *156/834* | *206/783* |  | *696/3260* |
| Adjusted model ^d^ | 1 | 0.97 (0.78 to 1.20) | 0.91 (0.73 to 1.13) | 1.20 (0.98 to 1.48) | *0.115* | - ^e^ |
| **≥5% WC increase ^c^** | | | | | | |
| **Normal weight** |  |  |  |  |  |  |
| *n for cases/non-cases* | *603/1541* | 637/1502 | 632/1508 | 700/1440 |  | 2572/5991 |
| Adjusted model ^d^ | 1 | 1.09 (0.98 to 1.22) | 1.13 (1.01 to 1.27) | 1.27 (1.14 to 1.42) | *<0.001* | 1.05 (1.03 to 1.07) |
| **Overweight** |  |  |  |  |  |  |
| *n for cases/non-cases* | *513/1809* | *579/1741* | *574/1743* | *605/1708* |  | 2271/7001 |
| Adjusted model ^d^ | 1 | 1.12 (0.99 to 1.26) | 1.15 (1.02 to 1.30) | 1.27 (1.13 to 1.43) | *<0.001* | 1.06 (1.03 to 1.08) |
| **Obesity** |  |  |  |  |  |  |
| *n for cases/non-cases* | *176/820* | *223/776* | *231/768* | *245/754* |  | 875/3118 |
| Adjusted model ^d^ | 1 | 1.26 (1.03 to 1.54) | 1.31 (1.08 to 1.60) | 1.44 (1.19 to 1.76) | *<0.001* | 1.07 (1.04 to 1.11) |
| **≥5% BF increase ^c^** | | | | | | |
| **Normal weight** |  |  |  |  |  |  |
| *n for cases/non-cases* | *352/471* | *401/420* | *374/448* | *402/417* |  | *1529/1756* |
| Adjusted model ^d^ | 1 | 1.15 (1.00 to 1.33) | 1.07 (0.92 to 1.24) | 1.13 (0.98 to 1.30) | *0.236* | 1.02 (0.99 to 1.05) |
| **Overweight** |  |  |  |  |  |  |
| *n for cases/non-cases* | *317/560* | *319/557* | *319/554* | *337/535* |  | *1292/2206* |
| Adjusted model ^d^ | 1 | 1.01 (0.86 to 1.18) | 1.02 (0.87 to 1.19) | 1.11 (0.95 to 1.29) | *0.203* | 1.03 (0.99 to 1.06) |
| **Obesity** |  |  |  |  |  |  |
| *n for cases/non-cases* | *85/310* | *96/302* | *120/276* | *122/275* |  | *423/1163* |
| Adjusted model ^d^ | 1 | 1.11 (0.83 to 1.49) | 1.39 (1.05 to 1.84) | 1.44 (1.09 to 1.90) | *0.003* | 1.09 (1.03 to 1.15) |
| * 705 (3.11%) participants reported they were currently on low calorie diet. | | | | | | |
| BMI = Body Mass Index; WC = waist circumference; BF = Body fat | | | | | | |
| ^a^ Sex specific cut-offs for quarters of ultra-processed food consumption. | | | | | | |
| ^b^ Defined according to World Health Organization cut-offs (WHO, 2003). | | | | | |  |
| ^c^ Participants who had a 5% increase in BMI/WC/body fat from baseline to follow-up. | | | | | |  |
| ^d^ Adjusted for age (as timescale), sex, Index of Multiple Deprivation (quintile and missing category); physical activity (low, moderate, high, missing category), smoking status (never, previous, and current) and sleep duration (≤6h/d, 7-8h/d, ≥9h/d); | | | | | | |
| ^e^ Non-linear association in restricted cubic spline regression. | | | | | |  |
| ^α^ p value for linear trend across quartile of dietary contribution of ultra-processed foods. | | | | | |  |

| **Supplementary Table S6. Association between dietary contribution of ultra-processed food (% of total energy) and indicators of obesity in the UK Biobank cohort. With models using average annual household income instead of Index of Multiple Deprivation (equivalent of Table 2).** | | | | | | |
| --- | --- | --- | --- | --- | --- | --- |
|  | **Ultra-processed food consumption (% of total energy)** | | | | | |
|  | **Sex-specific quarters ^a^** | | | |  | **Continuous (10% increase in the consumption)** |
|  | **1** | **2** | **3** | **4** |  |  |
|  | *HR (95% CI)* | | | | *p for trend^α^* | *HR (95% CI)* |
| **For being obese** ^b^ |  |  |  |  |  |  |
| Model 1 ^c,d^ | 1 | 1.19 (0.98 to 1.44) | 1.15 (0.95 to 1.39) | 1.58 (1.31 to 1.89) | *<0.001* | 1.11 (1.07 to 1.15) |
| Model 2 ^c,e^ | 1 | 1.21 (1.00 to 1.47) | 1.16 (0.96 to 1.41) | 1.60 (1.33 to 1.92) | *<0.001* | 1.11 (1.07 to 1.15) |
| Model 3 ^c,f^ | 1 | 1.45 (0.85 to 2.46) | 1.11 (0.63 to 1.96) | 1.84 (1.10 to 3.07) | *0.045* | 1.10 (0.99 to 1.22) |
| **For high WC ^g^** |  |  |  |  |  |  |
| Model 1 ^c,d^ | 1 | 1.15 (1.01 to 1.31) | 1.22 (1.07 to 1.39) | 1.38 (1.22 to 1.58) | *<0.001* | 1.07 (1.05 to 1.10) |
| Model 2 ^c,e^ | 1 | 1.16 (1.02 to 1.32) | 1.22 (1.07 to 1.40) | 1.39 (1.22 to 1.58) | *<0.001* | 1.07 (1.05 to 1.10) |
| Model 3 ^c,f^ | 1 | 1.17 (1.02 to 1.33) | 1.20 (1.05 to 1.37) | 1.29 (1.14 to 1.47) | *<0.001* | 1.06 (1.03 to 1.08) |
| **For having a ≥5% BMI increase ^h^** |  |  |  |  |  |  |
| Model 1 ^c,d^ | 1 | 1.06 (0.97 to 1.16) | 1.04 (0.95 to 1.14) | 1.27 (1.16 to 1.38) | *<0.001* | - ^i^ |
| Model 2 ^c,e^ | 1 | 1.07 (0.97 to 1.17) | 1.05 (0.96 to 1.16) | 1.28 (1.17 to 1.40) | *<0.001* | - ^i^ |
| Model 3 ^c,f^ | 1 | 1.07 (0.97 to 1.17) | 1.05 (0.96 to 1.15) | 1.28 (1.17 to 1.40) | *<0.001* | - ^i^ |
| **For having a ≥5% WC increase ^h^** |  |  |  |  |  |  |
| Model 1 ^c,d^ | 1 | 1.14 (1.06 to 1.23) | 1.18 (1.09 to 1.27) | 1.30 (1.21 to 1.40) | *<0.001* | 1.06 (1.04 to 1.07) |
| Model 2 ^c,e^ | 1 | 1.14 (1.06 to 1.23) | 1.18 (1.10 to 1.27) | 1.31 (1.21 to 1.41) | *<0.001* | 1.06 (1.04 to 1.07) |
| Model 3 ^c,f^ | 1 | 1.14 (1.05 to 1.22) | 1.18 (1.10 to 1.27) | 1.35 (1.25 to 1.45) | *<0.001* | 1.06 (1.05 to 1.08) |
| **For having a ≥5% BF increase ^h^** |  |  |  |  |  |  |
| Model 1 ^c,d^ | 1 | 1.08 (0.98 to 1.20) | 1.08 (0.98 to 1.19) | 1.15 (1.04 to 1.27) | *0.008* | 1.03 (1.01 to 1.05) |
| Model 2 ^c,e^ | 1 | 1.09 (0.99 to 1.20) | 1.08 (0.98 to 1.19) | 1.16 (1.05 to 1.28) | *0.005* | 1.03 (1.01 to 1.05) |
| Model 3 ^c,f^ | 1 | 1.07 (0.97 to 1.18) | 1.07 (0.97 to 1.18) | 1.16 (1.05 to 1.28) | *0.004* | 1.03 (1.01 to 1.05) |
| BMI = Body Mass Index; WC = waist circumference; BF = Body fat | | | | | | |
| Mean follow-up times were 5.6 for obesity (97,090 person-years), 5.6 for high waist circumference (91,380 person-years), 5.8 for having a ≥5% BMI increase (119,108 person-years), 5.8 for having a ≥5% WC increase (121,067 person-years), and 1.8 for having a ≥5% body fat increase (17,660 person-years), | | | | | | |
| ^a^ Sex specific cut-offs for quarters of ultra-processed food consumption - ranged from 25.5% of total energy intake (1st quartile) to 71.5% (5th quartile). | | | | | | |
| ^b^ Defined as Body Mass Index ≥30 kg/m^2^ (World Health Organization, 2003). | | | | | |  |
| ^c^ Age used as timescale in the Cox models. | | | | | |  |
| ^d^ Model 1: adjusted for sex and average annual household income. | | | | | |  |
| ^e^ Model 2: adjusted for Model 1 + physical activity (low, moderate, high, missing category), smoking status (never, previous, and current), and sleep duration (≤6h/d, 7-8h/d, ≥9h/d); | | | | | | |
| ^f^ Model 3: adjusted for Model 1 + Model 2 + BMI, WC or BF at baseline (according to the outcome); | | | | | |  |
| ^g^ Defined as waist circumference ≥102/88 cm for men and women, respectively (World Health Organization, 2008). | | | | | |  |
| ^h^ Participants who had a 5% increase in BMI/WC/body fat from baseline to follow-up. | | | | | |  |
| ^e^ Non-linear association in restricted cubic spline regression. | | | | | |  |
| ^α^ p value for linear trend across quartile of dietary contribution of ultra-processed foods. | | | | | |  |

| **Supplementary Table S7. Association between dietary contribution of ultra-processed food (% of total energy) and BMI, waist circumference and body fat according to the BMI status at baseline in the UK Biobank cohort. With models using average annual household income instead of Index of Multiple Deprivation (equivalent of Table 3).** | | | | | | |
| --- | --- | --- | --- | --- | --- | --- |
|  | **Ultra-processed food consumption (% of total energy)** | | | | | |
|  | **Sex-specific quarters ^a^** | | | | | **Continuous (10% increase in the consumption)** |
|  | **1** | **2** | **3** | **4** |  |  |
| **BMI status at baseline ^b^** | *HR (95% CI)* | | | | *p for trend^α^* | *HR (95% CI)* |
| **≥5% BMI increase ^c^** | | | | | | |
| **Normal weight** |  |  |  |  |  |  |
| Adjusted model ^d^ | 1 | 1.01 (0.87 to 1.16) | 0.99 (0.85 to 1.14) | 1.28 (1.12 to 1.47) | *0.001* | - ^e^ |
| **Overweight** |  |  |  |  |  |  |
| Adjusted model ^d^ | 1 | 1.16 (1.00 to 1.34) | 1.11 (0.96 to 1.29) | 1.36 (1.19 to 1.57) | *<0.001* | - ^e^ |
| **Obesity** |  |  |  |  |  |  |
| Adjusted model ^d^ | 1 | 0.96 (0.78 to 1.19) | 0.92 (0.75 to 1.14) | 1.19 (0.97 to 1.46) | *0.123* | - ^e^ |
| **≥5% WC increase ^c^** | | | | | | |
| **Normal weight** |  |  |  |  |  |  |
| Adjusted model ^d^ | 1 | 1.10 (0.99 to 1.23) | 1.13 (1.02 to 1.27) | 1.28 (1.15 to 1.43) | *<0.001* | 1.05 (1.03 to 1.07) |
| **Overweight** |  |  |  |  |  |  |
| Adjusted model ^d^ | 1 | 1.11 (0.99 to 1.25) | 1.16 (1.03 to 1.30) | 1.28 (1.14 to 1.44) | *<0.001* | 1.06 (1.03 to 1.08) |
| **Obesity** |  |  |  |  |  |  |
| Adjusted model ^d^ | 1 | 1.21 (1.00 to 1.47) | 1.28 (1.06 to 1.55) | 1.42 (1.18 to 1.72) | *0.001* | 1.08 (1.04 to 1.12) |
| **≥5% BF increase ^c^** | | | | | | |
| **Normal weight** |  |  |  |  |  |  |
| Adjusted model ^d^ | 1 | 1.16 (1.00 to 1.34) | 1.09 (0.94 to 1.25) | 1.15 (1.00 to 1.33) | *0.120* | 1.02 (0.99 to 1.05) |
| **Overweight** |  |  |  |  |  |  |
| Adjusted model ^d^ | 1 | 1.01 (0.87 to 1.18) | 1.04 (0.89 to 1.21) | 1.13 (0.97 to 1.31) | *0.123* | 1.03 (1.00 to 1.06) |
| **Obesity** |  |  |  |  |  |  |
| Adjusted model ^d^ | 1 | 1.09 (0.81 to 1.45) | 1.38 (1.05 to 1.82) | 1.44 (1.09 to 1.90) | *0.003* | 1.09 (1.04 to 1.15) |
| BMI = Body Mass Index; WC = waist circumference; BF = Body fat | | | | | | |
| ^a^ Sex specific cut-offs for quarters of ultra-processed food consumption. | | | | | |  |
| ^b^ Defined according to World Health Organization cut-offs (WHO, 2003). | | | | | |  |
| ^c^ Participants who had a 5% increase in BMI/WC/body fat from baseline to follow-up. | | | | | |  |
| ^d^ Adjusted for age (as timescale), sex, average annual household income, physical activity (low, moderate, high, missing category), smoking status (never, previous, and current) and sleep duration (≤6h/d, 7-8h/d, ≥9h/d); | | | | | | |
| ^e^ Non-linear association in restricted cubic spline regression. | | | | | |  |
| ^α^ p value for linear trend across quartile of dietary contribution of ultra-processed foods. | | | | | |  |
